# Stress, Prevention, and Resilience among First Responders

**DOI:** 10.3390/ijerph20247174

**Published:** 2023-12-13

**Authors:** Konstantinos Papazoglou

**Affiliations:** 1 ProWellness Inc., Woodbridge, ON L4H 0A2, Canada; dr.konstantinos@prowellnessinc.com or dr.konstantinos@aya.yale.edu; 2 The POWER Project, Woodbridge, ON L4H 0A2, Canada

This Special Issue [1] introduces and examines various topics on first responder’s stress. First responders and public safety personnel such as police officers, correctional officers, firefighters, and paramedics worldwide are responsible for assisting the public in crisis intervention and prevention. A career as a first responder is demanding and has the potential for burnout, stress, PTSD, suicide risk, and various mental health issues due to job demands. Therefore, first responders’ well-being should be a priority to ensure the more effective and safe execution of responsibilities that affect both first responders and the public. Moral risks and moral injury in police work are also examined to provide a deeper understanding of moral risk and its constituents to aid police agencies in addressing this issue within their department. The importance of addressing moral risk covers some significant concerns. Police officers who experience moral risk or moral injury are at an increased risk of PTSD, lapses in moral judgment, and the relaxation of ethical principles. Organizations’ need for change in policing culture is recommended. Creating an organization that supports first responders can also increase job satisfaction as the employee can feel a strong sense of unification. In a situation where a first responder’s mental health is compromised, they could experience significant difficulty in performing or not performing their duties adequately, which, in turn, could compromise the safety and well-being of the public. In line with this assumption, we need to strive to understand the challenges and issues that first responders face to assist them with organizational, operational, and social stressors by implementing practical training, policies, and education.

The uniqueness of this Special Issue is attributed to researchers specializing in first responder stress management and mental health issues worldwide. As first responders have similarities in their experiences around the world, it is imperative that we have a heterogeneous understanding of the challenges they face and the ways they can be addressed. In addition, many first responders experience burnout on the job. Rosca et al. (2021) examined burnout through the Job Demands-Resources (JD-R) model and the S = self-determination theory in firefighters. They asserted that the meaning of work was buffered between job demands and exhaustion. Deriving personal meaning from work was associated with lower levels of fatigue. This assertion follows a logical explanation because finding meaning in work equates with purpose. In addition, a 2021 study by the same author investigated the Dark Triad (psychopathy, narcissism, and Machiavellianism) of firefighters and found altruism, honesty, and courage to mediate an association between psychopathy and risk-taking. She argued for employing psychological testing to screen out potential employees prone to risk-taking and employing organizational training programs that could facilitate honesty, altruism, and courage. Organizational issues can contribute to the stress that first responders experience. Therefore, it is vital to examine how an organization can assist in helping first responders, as they act as a primary source of support.

Organizational stress is also an essential element of mental health concerns in first responders. Factors such as staff shortages, inconsistent leadership styles, shift work, sleep, stigma, and public scrutiny have all been identified to play a major role in the development of mental health disorders even after the influence of potentially psychologically traumatic events is statistically controlled, which points to the need for improved organizational and operational stressors through much-needed support. In line with this assertion, Jessiman-Perreault et al., 2021 support structural policy changes to integrate interpersonal support programs that aim to increase coping and resiliency. Furthermore, Blumberg et al. (2019) corroborate that law enforcement agencies are responsible for incorporating psychological skills to meet the needs and challenges that police officers face in the line of duty. Cognitive, emotional, social, and moral skills may all be incorporated into training for a non-authoritative stand into an adult learning model that fosters positive psychological skills to create a safe and successful police culture and working environment. Incorporating resilience training and building can significantly and positively affect the mental health of first responders. Resilience building for first responders has been at the forefront of researchers’ and clinicians’ studies. Gaining a thorough understanding of the factors contributing to resiliency could benefit first responders personally and communally. Katzman et al. (2021) evaluate the Extension for Community Outcomes (ECHO) program for first responders and identifies that although overall stress levels were not reduced, participants had increased confidence in using psychological first aid, recognizing and managing colleagues who needed support, and making time for self-care. Resilience building is the multifaceted ranking from prevention or moral risk to ameliorating PTSD symptomatology. Sleep quality is also an essential factor that shows a bidirectional relationship between mental health disorders and work impairment; therefore, targeting sleep discrepancies is a crucial component of mental well-being in first responders. Workplace policies that integrate sleep hygiene in their training are deemed beneficial in a study by Angehrn et al. (2020). Evidence-based access to mental health support and educational self-care strategies can help facilitate well-being. Access can come in many forms, including organizational, social, and online.

Online resources have become even more prominent nowadays; many people use online services to help them in myriad ways, from looking up a recipe to diagnosing an illness. First responders are no different in that they search the world wide web for mental health support. Killip et al. (2020) examines the quality of PTSD resources generated online and how promoting recommendations align with current evidence-based interventions. A discrepancy indicated the varying quality of comprehensiveness and misalignment with current best practices. Online resources should be assessed to present accurate and reliable information when first responders seek online mental health services. Finally, organizational prevention suggestions are made to assist with systemic factors and moral risks addressing operational readiness and preventing undesirable outcomes. Job satisfaction, with a focusing on altruism, honesty, and courage, is examined to mediate psychopathy and risk-taking in firefighters. Interventions with first responders such as psychological first aid, critical incident debriefing, the examination of moral distress, crisis management strategies, and self-care skills are all implicated in the well-being of first responders.

Future research and clinical practice, as well as organizational policies and structures, should address the specific needs of first responders. All the above topics should be holistically examined as they interplay to foster or alleviate mental health issues. Providing psychological skills-based training and the translation of mental health risks and psychoeducational workshops can positively enhance first responders’ mental health. Starting with primary resources such as accessible online support that is high quality and easy to understand could assist first responders in their attempt for self-help. The requirement of police candidates to obtain some type of certification independently before employment could also have a positive effect. Police organizations could benefit from preparing new workers prior to the commencement of the academy and implementing practices and training that focus on not only the physical and operational duties carried out but also the psychological ones.

Candidates may study and practice topics such as procedural justice, community relations, stress management, and psychological skills.

Officer well-being and ethics can be emphasized when recruiting and in hiring, training, supervision, and promotion methods. It necessitates police commanders who are prepared to question the established quo. Nonetheless, these crucial improvements have the ability to enhance officer safety and relations between police organizations and the community in the long run. Policymakers should look for methods to reduce occupational pressures to support first responders’ mental health, such as making workplaces more psychologically safe. A practical action plan to treat and prevent PTSD and other mental health illnesses would also rely on adjustments to lessen organizational and operational pressures. Focusing on leadership training and support, greater organizational participation, stigma reduction, improved sleep, and social support could yield tremendous benefits.
